# Individual and conjunctive operation of tidal lagoons along the west coast of the UK

**DOI:** 10.1007/s40722-025-00420-x

**Published:** 2025-09-01

**Authors:** Bin Guo, Reza Ahmadian, Roger A. Falconer

**Affiliations:** 1 School of Engineering, Cardiff University, The Parade, Cardiff, CF24 3AQ UK; 2 National Oceanography Centre, 6 Brownlow Street, Liverpool, L3 5DA UK

**Keywords:** Tidal energy, Tidal range scheme, Hydrodynamic impact, Conjunctive operation

## Abstract

The conjunctive operation of multiple Tidal Range Schemes (TRSs) along a coastline, particularly where tidal phasing is appreciable, may partially offset the power output intermittency when TRSs are considered in isolation. This study investigates the individual and conjunctive operations of three prospective tidal range schemes: North Wales Tidal Lagoon (NWTL), West Somerset Lagoon (WSL) and Swansea Bay Lagoon (SBL). Each lagoon was assessed individually and then conjunctively to explore their potential interaction on the hydrodynamic impact and power output. Under individual operation, the tidal velocity magnitude across the NWTL impoundment typically increased by 0.1–0.5 m/s, in contrast to the decreased maximum current velocity observed within the WSL and SBL basins. The analysis of open boundary placement in lagoon modelling underscores the importance of employing a sufficiently large model domain for accurate simulation of TRSs. In the conjunctive operation of WSL and NWTL, as well as WSL and SBL, interactions between the lagoons were found to be minor. However, the duration with no power generation for lagoons running independently was reduced from 2.5 to 3.75 h to less than 1.5 h, demonstrating the feasibility of delivering near-continuous tidal power through the conjunctive operation of WSL and NWTL. This study is the first to explore the hydrodynamic interactions of conjunctive tidal lagoon operations in the UK, demonstrating their mutual impacts and the potential of coordinated operation to reduce intermittency.

**Supplementary Information:**

The online version contains supplementary material available at 10.1007/s40722-025-00420-x.

## Introduction

Electricity generation from tidal range schemes (TRSs), which utilises the energy from large tidal elevation differences, is highly predictable and abundant across much of the western coast of the UK (Ahmadian et al. 2010; Neill et al. 2018). However, TRSs have some inherent issues that have hindered their development to date. The high upfront construction costs, relatively long construction time and the potential environmental impacts are key challenges relating to the delivery of TRSs (Waters and Aggidis 2016; Hooper and Austen 2013; Falconer et al. 2020; Kadiri et al. 2014; Hanousek et al. 2023). Additionally, the power output from TRSs is inherently intermittent, fluctuating with both the semi-diurnal tides that occur twice daily and the fortnightly spring–neap cycle. This variability complicates grid management and amplifying challenges to deliver a stable power supply (Mackie et al. 2020; Vazquez and Iglesias 2015; Todeschini 2017).

Efforts to establish a continuous power supply from TRSs have been made but have often proved impractical as evidenced by attempts using linked-basin TRSs (Angeloudis et al., 2020) and pumped storage systems (MacKay 2007). However, if two or more TRSs are located such that a reasonable interval exists in the tidal phase between the sites, the operation of the schemes can be optimised by treating as a coordinated system. Adopting this approach, a network of combined TRSs can offer the flexibility to offset the variability in power output from individual schemes, moving closer to delivering base-load power (Neill et al. 2018). In addition, at a single tidal energy-rich site, the operation of multi-TRSs could be designed to extract the maximum energy from the available tidal range. Therefore, the conjunctive operation of multiple TRSs has been proposed as a more effective regional tidal energy deployment strategy compared to a single scheme (Neill et al. 2018; Cornett et al. 2013). It is worth noting that whilst Tidal Range Schemes (TRSs) encompass both tidal lagoons and barrages, differing primarily in their degree of water blockage, these two forms generally follow the same operational procedures. Therefore, both can be integrated into the same conjunctive operation system. This paper, however, specifically examines tidal lagoon projects due to their increasing relevance and attention in current research.

However, combined hydrodynamic impacts may be expected to arise from the conjunctive operation of multiple TRSs, potentially affecting both the environment and the performance of the individual lagoons in electricity generation (Wilson et al. 2012a, b; Wolf et al. 2009; Cornett and Cousineau 2011; Angeloudis and Falconer 2017). Even minor changes in tidal range can lead to significant cumulative effects on electricity output due to the non-linear relationship between power generation and tidal range (Angeloudis and Falconer 2017). These effects are particularly relevant when TRSs are located within the same channel or estuary. Therefore, it is essential to evaluate the potential interactions between TRSs from multiple perspectives.

Angeloudis and Falconer (2017) modelled the joint operation of Swansea Bay Lagoon, Cardiff Lagoon and Newport Lagoon, and assessed the combined hydropower and environmental impacts. From this study, a cumulative hydrodynamic impact was predicted in the proximity of Swansea Bay Lagoon and in the Severn Estuary, wherein changes in water levels and tidal velocity were observed. Wolf et al. (2009) simulated tidal barrages located in five major estuaries along the west coast of the UK, including the Severn, Dee, Mersey, Morecambe Bay and Solway estuaries. An insignificant far-field impact was observed, except for a potential predicted 10% increase in the tidal range along the east coast of Ireland, thereby increasing potential coastal flood risk. Cornett and Cousineau (2011) modelled the joint operation of three offshore lagoons and three coastal lagoons in the Bay of Fundy, Canada. Their findings showed that the combined operation of all six lagoons induced significant hydrodynamic changes—specifically, an approximate 5.5 cm increase in the high-water level of Boston tides, compared to only a 1.4 cm increase caused by a single coastal lagoon.

Most of the existing TRSs have been considered in isolation and operated individually, without considering the conjunctive operation of the scheme with other TRSs, or other renewable energy projects (Waters and Aggidis 2016; Cho et al. 2012; Rtimi et al. 2021). Therefore, further research is needed to explore the feasibility of this coordinated operational method, and to evaluate the potential interactions between TRSs on electricity generation and the coastal environment. This is particularly important because even minor changes in tidal range can lead to significant cumulative effects on electricity generation, due to the non-linear relationship between power output and tidal range (Angeloudis and Falconer 2017). Furthermore, the interactions and accumulated impacts of tidal lagoons and barrages cannot be simply added together to estimate their cumulative impacts. This is because any hydrodynamic changes, especially water level changes, could have a direct impact on the operation of another lagoon and vice versa. For this purpose, the first systematic study of the potential hydrodynamic interactions between tidal lagoons is reported in this paper, by modelling and analysing both their individual and conjunctive operations. Moreover, the impact of the open boundary location on lagoon modelling was explored, emphasising the importance of using an appropriate hydrodynamic model as the basis for accurate lagoon modelling impact predictions. The results of this research can serve as a useful reference for the study of tidal range systems in other regions.

## Tidal lagoon cases

**Fig. 1:**
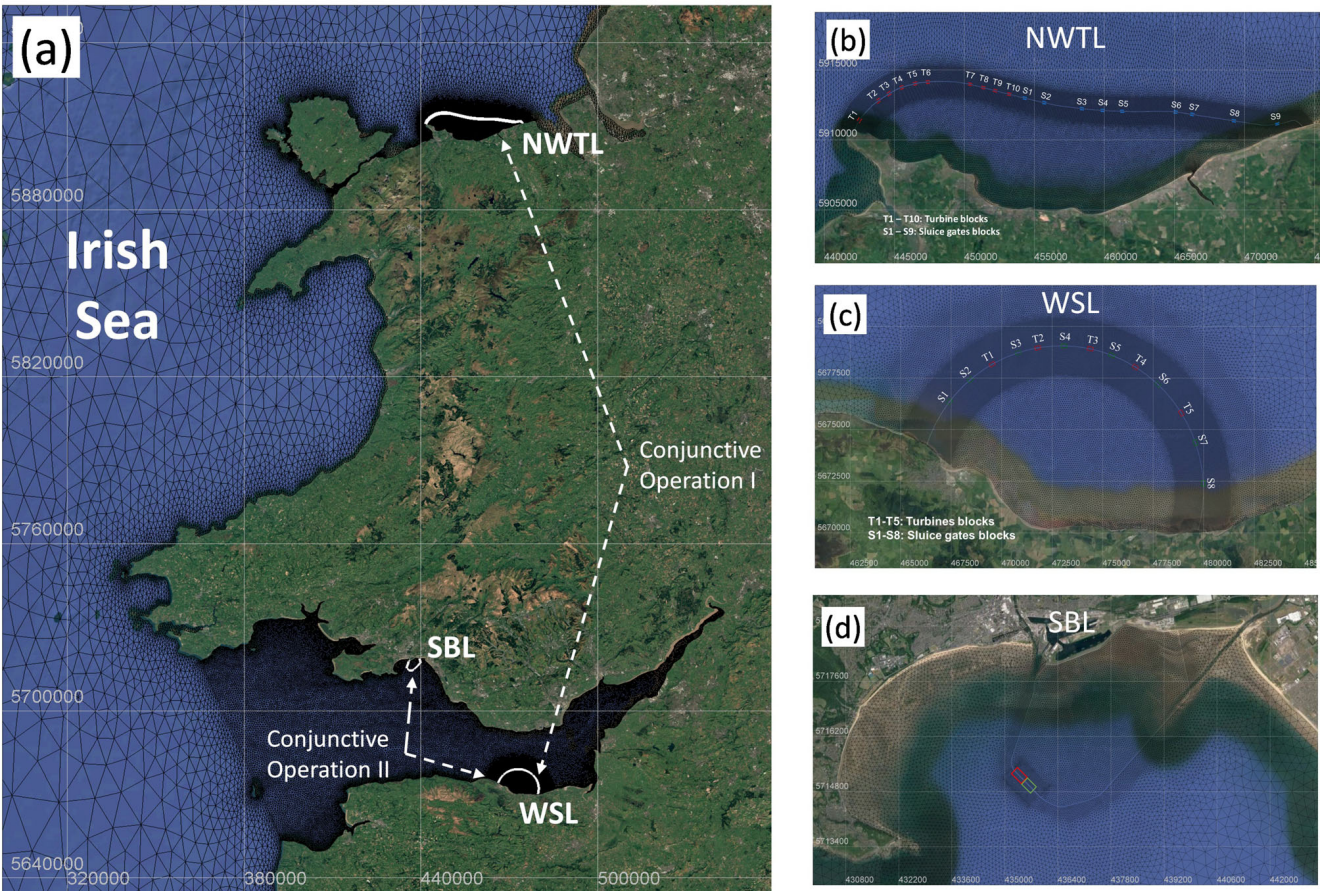
Locations and configurations of the tidal lagoons studied in this research, including the two lagoon combinations (Combinations I and II) considered for conjunctive operation. (Background satellite imagery from Google Map)

The power output and the environmental impact of three lagoons proposed by three different companies are considered in this study, namely North Wales Tidal Lagoon (NWTL), West Somerset Lagoon (WSL) and Swansea Bay Lagoon (SBL). Each of them has its unique design features and can also represent the state-of-the-art TRSs design, as shown in Fig. 1. One conjunctive operation combination considered is the NWTL and the WSL, which exhibit a tidal phase difference of more than four hours between the two lagoon locations, thereby helping to partially offset the variability in power output from each lagoon (Neill et al. 2018; Mackie et al. 2020). Another lagoon combination considered is WSL and SBL, both situated in the Bristol Channel, where their proximity and tidal resonance characteristics may influence hydrodynamic interactions (Ma and Adcock 2020). Given the numerous lagoon proposals in this region, it would be insightful to simulate multi-lagoon operations and explore their collective impact (Waters and Aggidis 2016).

The tidal range along the North Wales coast exceeds 8 m during mean spring tides (Angeloudis et al. 2015), one of the highest in the UK, except for that in the Severn Estuary and Bristol Channel. The marine topography in this area consists of shallow waters adjacent to deeper waters, which is ideal for the construction of TRSs with minimised construction costs, making the North Wales Coast an attractive site for a tidal lagoon construction. The early-stage planning area of the lagoon was planned to span from Llandudno to Rhyl, with a breakwater stretching over 30 km in length and a water impoundment area of 150 km^2^, such shape of the NWTL along the coastline was designed to protect vulnerable communities from sea flooding (North Wales Tidal Energy Ltd 2020). Based on power prediction results from a 0D research study (Xue et al. 2020), 150 turbines with capacity of 3 GW were predicted to give the optimum power output of NWTL, sited in 10 blocks along the perimeter wall. Nine blocks of sluice gates were also included, to contribute to maximising the electricity generation and reducing water quality changes, with a total sluicing area of 20,000 m^2^. The turbines and sluice gates of the NWTL scheme were distributed primarily based upon the regional bathymetric features, as seen in Fig. 1(b), but also taking account of the expected reduced environmental impacts likely at these locations.

West Somerset Lagoon and Swansea Bay Lagoon are located in Bristol Channel with spring tidal range of 12.2 m (Ma and Adcock 2020). The WSL proposal includes a semi-circular impoundment with a length of 22 km, and an enclosed area of approximately 80 km^2^ as presented in Fig. 1(c). More details about WSL are described in Mackie et al. (2020) and Guo et al. (2021). Swansea Bay Lagoon (Fig. 1(d)), the closest scheme to commercial viability, has been extensively studied. Although this proposal was terminated, it could still act as a benchmarking (Čož et al. 2019; Angeloudis et al. 2018), and can be used for potential TRSs interaction study.

## Methods

### Hydrodynamic modelling

The TELEMAC model suite was selected as the numerical tool for this research study based on its wide range of marine and coastal energy modelling applications, triangular mesh and easily accessible open source code. The equations and algorithms of TELEMAC can be found in many other publications (Hervouet 2007; Guo et al. 2021; Haverson et al. 2017). Based on the location of the TRSs in this research study, two hydrodynamic models were developed and validated: the Continental Shelf (CS) model and the Severn Estuary and Bristol Channel (SEBC) model.

**Fig. 2:**
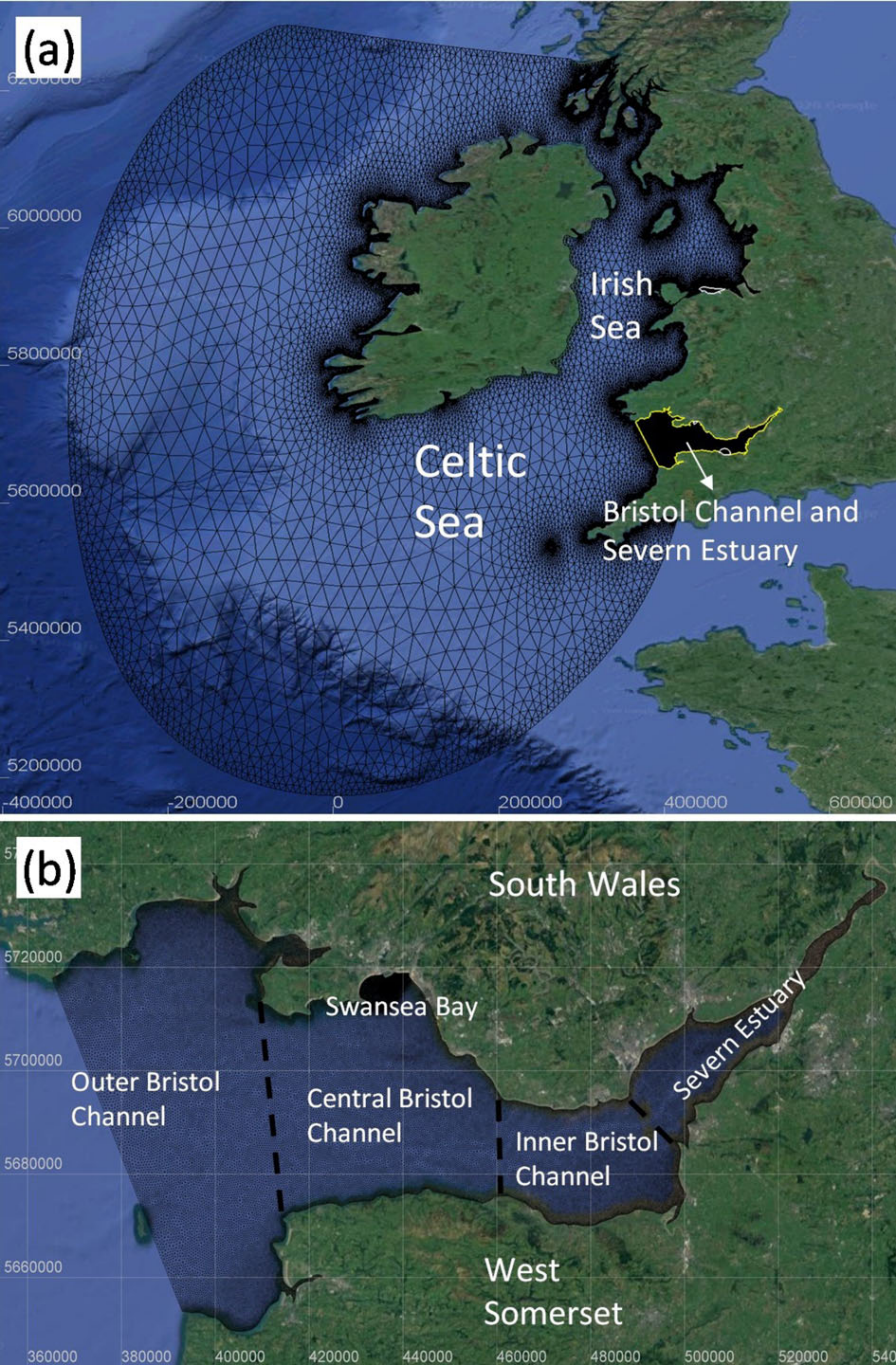
**a** Domain of the Continental Shelf (CS) model; (**b**) Domain of the Severn Estuary and Bristol Channel (SEBC) model, as well as its corresponding geographical division

The Continental Shelf (CS) model was established to include the NWTL and WSL in the same modelling domain, covering the Irish Sea and most of the Celtic Sea, with its open boundary extended to the Continental Shelf and where the tides are effectively generated, as shown in Fig. 2(a). The open boundary of the CS model extended from Plymouth in southwest of England to the Isle of Mull, on the west coast of Scotland, as shown in Fig. 2(a). Most of the domains from the CS model utilised bathymetric data from the EMODnet bathymetry portal, with 1/24 of an arcminute resolution (approx. 75 m). Moreover, higher-resolution topographic data were utilised in areas of particular interest. Topographic data for the Bristol Channel and Severn Estuary, along with the region off the North Wales coast and across Liverpool Bay, were obtained from EDINA Digimap, with a finer resolution of 1 arcsecond (approx. 30 m).

The unstructured mesh of CS model was discretised with 134,291 nodes and 252,382 elements. The mesh resolution varied from 9 km near the open boundary to a maximum value of 35 km in the middle of the model domain; then it was reduced to 700 m along the coastline. Moreover, in the areas of interest, such as the Severn Estuary and Bristol Channel, Colwyn Bay, and Liverpool Bay, a higher mesh resolution was provided varying from 50 to 300 m as shown in Fig. 2(a). The seaward open boundary was driven by spatially varying time histories of tidal elevations and depth-averaged velocities from the TPXO7.2 database (Dushaw et al. 1997; Bourban et al. 2012). The CS model uses the classic k–ε turbulence model and a conjugate residual solver, which has been proven to be suitable for modelling tidal flow around obstructions in a macro-tidal estuary (Ji et al. 2020). The Courant number limitation was set to 1, and the time step was set to 10 s to ensure model accuracy and computational efficiency. After repeated runs to calibrate the bottom roughness, the Manning’s roughness coefficient of 0.025 was found to produce the most accurate validation. Moreover, four days of spin-up time was used in the CS modelling due to the size of the model domain. River discharges from key sources were included due to their potential impact on regional hydrodynamics: seven rivers in the Bristol Channel model (Taff, Usk, Wye, Severn, Avon, Parrett and Tawe), and four rivers in Colwyn Bay and Liverpool Bay (Dee, Mersey, Ribble and Colwyn). These data, provided as daily averages, were obtained from the UK National River Flow Archive.

The research study relating to the interaction between SBL and WSL was more focussed on the Severn Estuary and Bristol Channel. Thus, a hydrodynamic model covering the Severn Estuary and Bristol Channel (SEBC) was developed, as seen in Figs. 2(b), which satisfied the requirements of the research and enhanced the computational efficiency for a wide range of model simulations. Most of the model parameters were the same between the CS model and SEBC model. However, different boundary conditions were applied to the SEBC model. A time series of water levels was applied to the open boundary of the SEBC model, which was previously evaluated as being a stable and accurate representation of the open boundary conditions (Guo et al. 2020; 2021).

Hydrodynamic models covering the whole Bristol Channel have been used to simulate small TRSs in previous studies (Angeloudis et al. 2016; Čož et al. 2019; Xia et al. 2010a, b). However, it is reported that an open boundary within the continental shelf may amplify any perturbation associated with a TRS by exciting a resonant mode (Adcock et al. 2015). Thus, any model that simply held the same boundary condition for pre- and post-TRS scenarios may demonstrate discrepancies in the impacts on hydrodynamics of the region (Zhou et al. 2014; Rainey 2009). For this purpose, this study was extended to evaluate the impact of WSL on the hydrodynamic characteristics in the Bristol Channel based on predictions from the CS and SEBC models, which thereby includes investigating the effect of open boundary location on the impact of the lagoon in operation (Sect. 4.5).

### Tidal lagoon modelling

To simulate the TRS accurately, the conservative momentum method was applied by including the external momentum term in the TELEMAC system (Guo et al. 2021). The mesh decomposition method was also applied to split the water impoundment area from the sea external to the basin. The discharge and the calculated power through the turbines and sluice gates were coupled with a ramp function to ensure a smooth transition between the operation modes. It was concluded from a previous study that the optimised generation scheme with flexible start and end heads had noticeable benefits in terms of electricity generation and minimising the environmental impacts (Guo et al. 2021; Angeloudis et al. 2018). Thus, the optimised modes of operation achieved from the 0D model for each scheme were implanted into the lagoon operation modelling (Xue et al. 2020).

It was observed that the embankment of the NWTL and WSL spans the tide propagation direction in the coastal zone, with the turbine and sluice gate blocks distributed widely along the embankment. As a result, a significant consideration is that there is a non-negligible water level difference along the two tidal lagoons, as shown in Fig. 12. This spatial variation means that different turbine or sluice gate blocks may experience differing water levels difference, particularly where the turbines and sluice gates are located on two sides of the embankment. Such variations can affect the accuracy of simulations evaluating the operation efficiency of lagoons. Therefore, a delicate operation was applied in the modelling of WSL and NWTL, with each turbine and sluice gate block being operated individually in the model. The operation stage and the discharge for each turbine and sluice gate block were therefore determined by the water level difference across the hydraulic structure at each individual block. The details of the operation scheme are illustrated in Fig. 13.

The operation schemes of the NWTL were illustrated to demonstrate the functioning of tidal lagoons (Fig. 14 and Fig. 15). Two operational strategies were applied: optimised two-way generation with and without pumping, both incorporating a flexible generating head (Xue et al. 2020). Figure 14 presents the variations in water levels upstream and downstream of the impoundment, along with the corresponding water discharge and power output, to evaluate the performance of NWTL operations. Additionally, Fig. 15 displays representative instantaneous flow patterns and velocity magnitudes during two-way operation.

## Results

### CS hydrodynamic model validation

The model predictions using the Continental Shelf (CS) model were first validated against measured water level data at 12 tide gauges in the Irish Sea and Bristol Channel as presented in Table 4 in the appendix. The validation period was one month, ranging from 17/05/2012 00:00:00 to 16/06/2012 00:00:00. The coefficient of determination R^2^ and the root mean square error (RMSE) values obtained in comparing the computed results and observation data are provided using the following formulations:

1 $$ R^{2} \, = \,1 - \frac{{\mathop \sum \nolimits_{i = 1}^{n} \left( {X_{i} - Y_{i} } \right)^{2} }}{{\mathop \sum \nolimits_{i = 1}^{n} \left( {Y_{i} - \overline{Y}} \right)^{2} }} $$

2 $$ RMSE\, = \,\sqrt {\frac{1}{n}\mathop \sum \limits_{i = 1}^{n} \left( {X_{i} - Y_{i} } \right)^{2} } $$

where ${Y}_{i}$ is the observed value, $\overline{{Y }_{i}}$ is the average of the observed values, ${X}_{i}$ is the predicted value. The model validation shows good correlation between the model predictions and measured data, as demonstrated in Table 4 and Fig. 16. The R^2^ values for the water level comparisons at tidal gauge sites in the Irish Sea are all higher than 0.96. The average RMSE is 0.213 m, equivalent to approximately 3.2% of the average spring tidal range (~ 6.61 m), indicating a high level of accuracy in the tidal elevation simulation. The water levels in the Irish Sea region, such as Portpatrick, Port Erin, Liverpool, Llandudno and Holyhead, show close agreement between the model results and measured data. The RMSE values for the validation of water levels at Mumbles, Hinkley and Ilfracombe are slightly higher, but still acceptable bearing in mind the very high tidal ranges in the Bristol Channel.

Furthermore, the velocity magnitude and the direction predictions for the CS model were validated against ADCP data measured in Swansea Bay during April 2012 (Guo et al. 2021). A representative comparison of velocity is shown in Fig. 17, with an RMSE of 0.097 m/s and an R^2^ value of 0.77, relative to an average observed velocity magnitude of approximately 0.58 m/s. This indicates that the model also performs well in simulating tidal currents.

### Hydrodynamic impact of individual operation of NWTL and WSL using the CS model

**Fig. 3:**
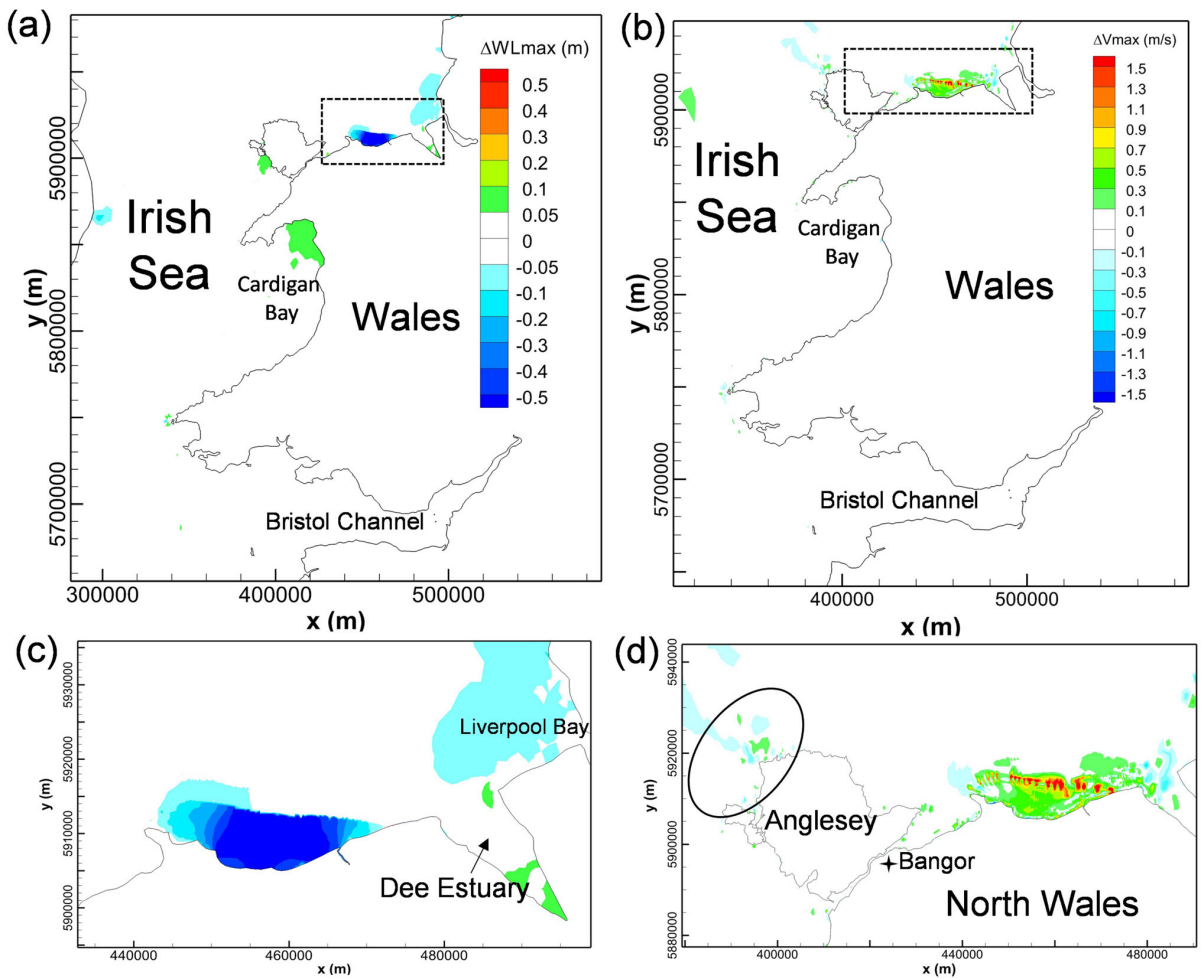
Cumulative impact of North Wales Tidal Lagoon on maximum water levels (left column) and maximum velocity (right column). The lower figures show a zoomed-in view of the North Wales coast region, as indicated by the dashed black line in (**a**) and (**b**)

Two-way operation of NWTL will cause a significant reduction in the high-water level inside the impoundment, as shown in Fig. 3(a)(c), which effectively reduces the flood risk along much of the North Wales coast from storm flooding and the long-term sea-level rise (Ahmadian et al. 2014). It is also observed that the NWTL would slightly reduce the high-water levels in Liverpool Bay, whilst slightly increasing the levels in the northern part of Cardigan Bay, with both variations ranging from 5—10 cm.

The impact of the NWTL operation on the tidal current speed is mainly concentrated in the near-field and within the impoundment basin, as shown in Fig. 3(b)(d). It is noticeable that the current speed increases significantly at the exit of the turbines and sluice gates due to the pronounced wakes. The strong wakes are found to be more noticeable around the sluice gates due to the shallower bathymetry in this region and the relatively large sluicing area.

The velocity in the landward areas inside NWTL also increased by typically 0.1–0.6 m/s. This phenomenon is inconsistent with previous research results from other TRS studies, where the current speed usually decreased in most regions away from the turbine and sluice gate wakes of the TRS impoundment (Guo et al. 2021; Angeloudis et al. 2016). The increased current speed in the impoundment waters of NWTL was predicted to be caused by the split layout of the turbines and sluice gates, which caused a convergence of the water stream along the east–west direction inside the lagoon basin. In addition, as highlighted in Fig. 3(b), the operation of NWTL would introduce small velocity increases and decreases on the northwest side of Anglesey, where the deployment of tidal stream turbines attracts extensive research interest due to the high tidal velocity in the region (Haverson et al. 2017; Lewis et al. 2015; Serhadlıoğlu et al. 2013). The observed pattern is likely due to local flow redistribution resulting from the lagoon’s influence on tidal phase and amplitude, which alters the velocity gradients in this hydrodynamically sensitive area.

**Fig. 4:**
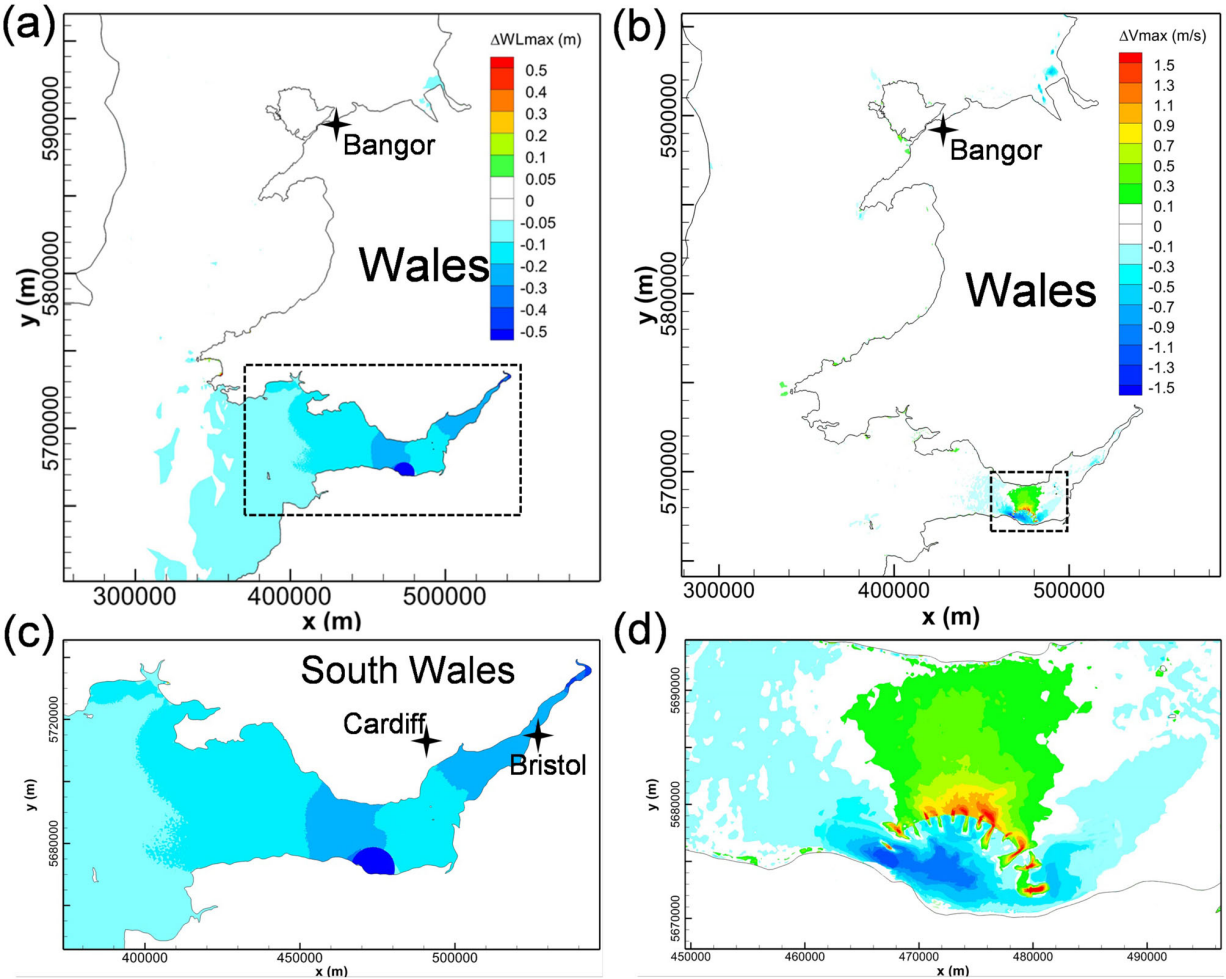
Cumulative impact of West Somerset Lagoon predicted in CS model on maximum water levels (left column) and maximum velocity (right column). The lower figures show a zoomed-in view of the Bristol Channel region, as indicated by the dashed black line in (**a**) and (**b**)

The hydrodynamic changes induced by WSL, including high-water level and the maximum tidal velocity changes, are shown Fig. 4(a)(c), showing that the impact of WSL operation on the high-water level extends to the mouth of the Bristol Channel, with the maximum water level in the outer Bristol Channel decreasing by 5–10 cm. The decrease in maximum water level becomes more noticeable in the landward direction, with a 10–20 cm reduction in the middle Bristol Channel and a 20–30 cm drop distributed in the inner Bristol Channel and the Severn Estuary. However, only minor changes were found outside of the Bristol Channel, in the Irish Sea and the Celtic Sea. Due to the relatively small scale of the basin, higher-resolution modelling would be desirable to predict these water level reductions more precisely, and particularly over the sensitive tidal wetlands in the Severn Estuary.

An increase in the velocity magnitudes of typically 0.2–1.3 m/s is predicted to occur in the inner Bristol Channel, whilst a slight decrease of 0.1–0.7 m/s occurred on both sides of WSL. The maximum velocity changes were mainly concentrated in the proximity of the WSL, particularly within the high-speed water jets through the turbines and sluice gates (Guo et al. 2021).

### Interactions between NWTL and WSL

**Fig. 5:**
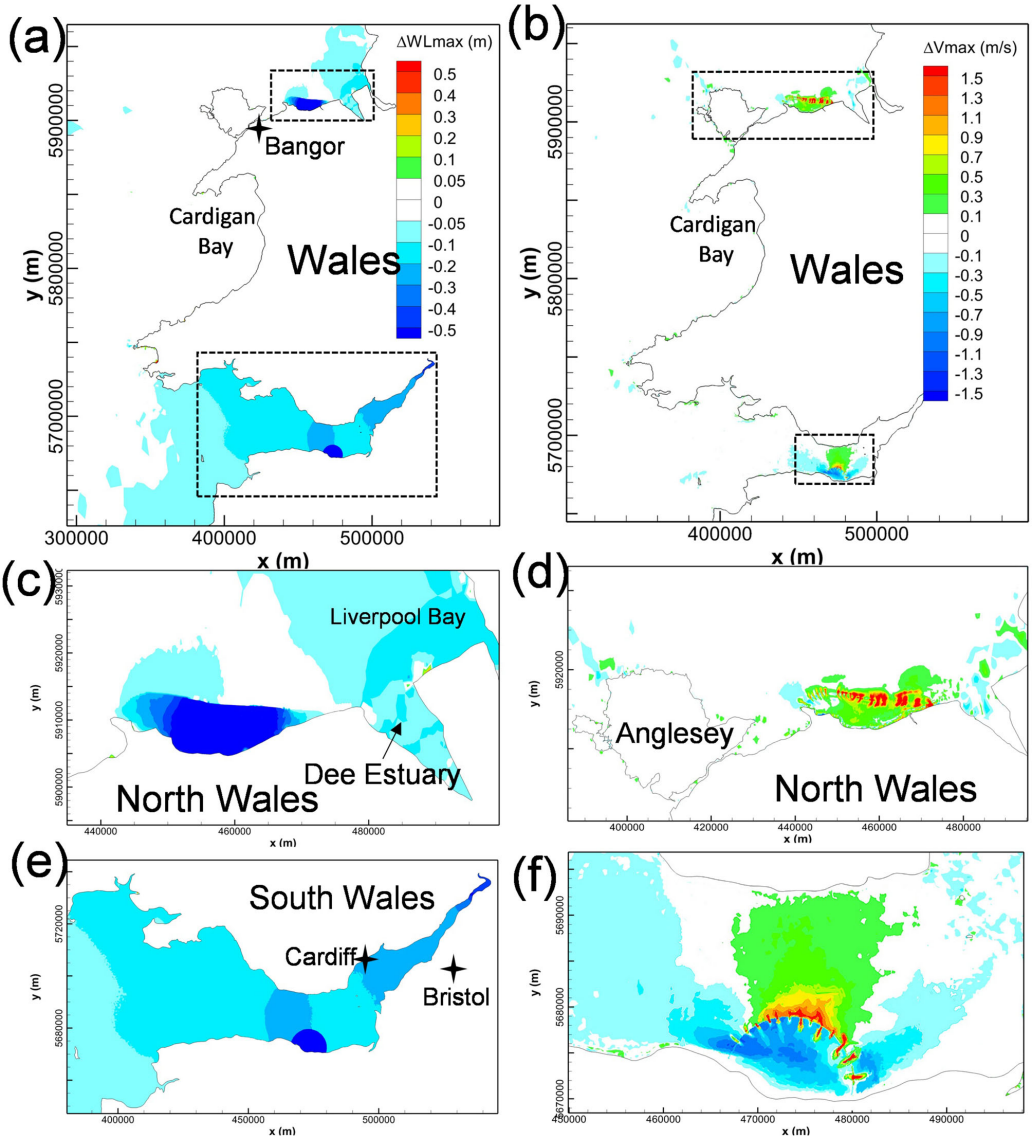
Cumulative impact of conjunctive operation of NWTL and WSL on maximum water levels (left column) and maximum velocity (right column). The lower figures show a zoomed-in view of the interest region, as indicated by the dashed black line in (**a**) and (**b**)

NWTL and WSL were then implanted into the CS model together and operated jointly to investigate their interaction and the cumulative impact on the surrounding waters, with the difference of maximum water level and velocity magnitude presented in Fig. 5.

By comparing the high-water level changes between Fig. 3, Fig. 4 and Fig. 5, the predicted interaction on the high-water levels between WSL and NWTL operating individually and conjunctively can be analysed. The operation of NWTL has a negligible impact on the high-water level near the WSL and vice versa. This demonstrates that the interaction between WSL and NWTL has a negligible impact on the near-field water level predictions of the other TRS. However, some effects emerge from the joint operation of the schemes. The increased water level in Cardigan Bay found with the individual operation of the NWTL is mitigated under the conjunctive operation of both lagoons, which might be a result of far-field interaction between WSL and NWTL as the 2–3 cm decreased high water level in Cardigan Bay caused by the WSL offset the increased water level result from the NWTL. Furthermore, in the conjunctive operation scenario, a larger area of showing a decrease of about 5–10 cm appeared in Liverpool Bay. Similar results were found for the maximum velocity changes by comparing Fig. 3(d), Fig. 4(d) and Fig. 5(d)(f). Negligible interactions between NWTL and WSL are observed for maximum tidal flows, with changes in tidal currents being mainly confined within the near-field of the individual lagoons.

**Fig. 6:**
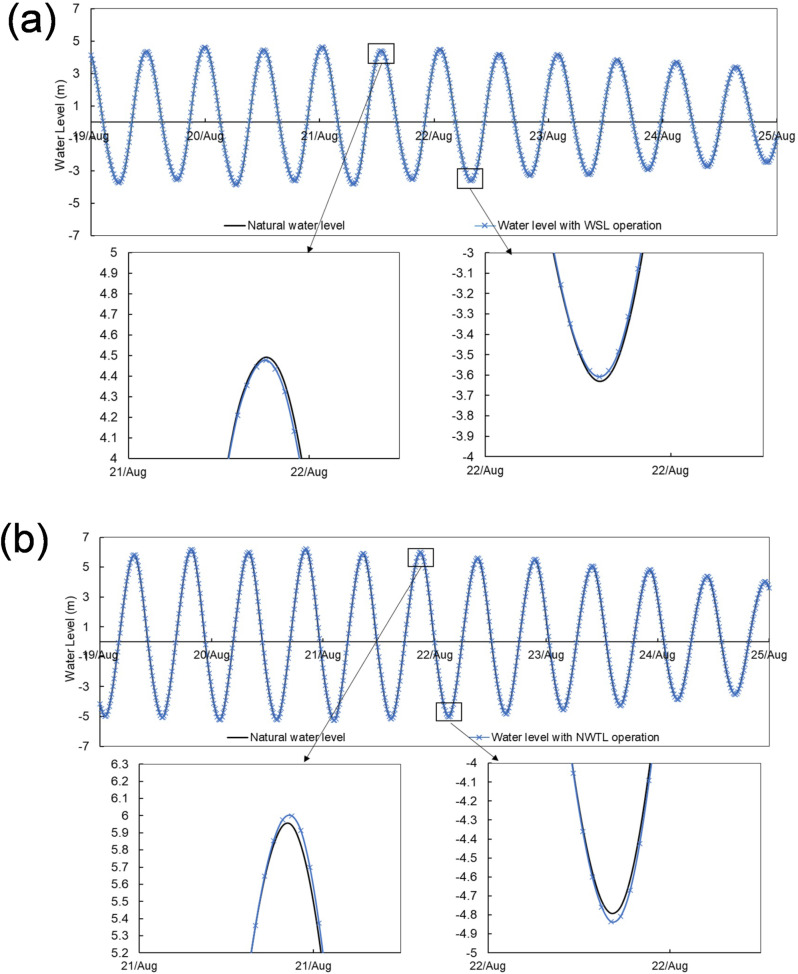
Water level (WL) changes in: **a** NWTL planned area for pre- and post-WSL operation; **b** WSL planned area for pre- and post-NWTL operation

The changes in water level in the Colwyn Bay area, where the NWTL is located, for pre- and post-WSL scenarios, are presented in Table 1 and Fig. 6(a). From these results, it is observed that the operation of the WSL slightly reduces the tidal range in the planned NWTL area by a few centimetres. Correspondingly, Table 1 and Fig. 6(b) show the water level changes for the proposed WSL impoundment for pre- and post-NWTL construction scenarios. The high-water level was predicted to increase by up to about 9 cm during spring tides, whilst the low water level decreased by a similar value, resulting in an enlarged tidal range in the WSL impoundment area.

**Table 1:** Water level changes for pre- and post-lagoon scenarios

Water level variation	HW of spring tide	LW of spring tide		HW of neap tide	LW of spring tide
WL change along the Bristol Channel with the presence of NWTL	+ 2–9 cm		−1–7 cm	+ 1–4 cm	−1–3 cm
WL change along the North Wales coast with the presence of WSL	−1–5 cm		+ 0–4 cm	−0–3 cm	+ 0–2 cm

Table 2 shows that the interactions between WSL and NWTL had slightly opposing impacts on the electricity output from each TRS. The operation of WSL decreased the electricity output of NWTL by about 0.48% and 0.41% for a flexible two-way generation without pumping and with pumping, respectively. This result is consistent with the water level changes in the NWTL water impoundment area for pre- and post-WSL conditions, as shown in Fig. 6(a) and Table 1 i.e. that the decrease in tidal range results in a slight decrease in the electricity output. Likewise, the operation of NWTL could increase the electricity output of WSL by about 1.6% and 1.1% for the two operation schemes as a result of the raised tidal range in the inner Bristol Channel.

**Table 2:** Electricity outputs of WSL and NWTL for individual and conjunctive operation in one entire tidal cycle, considering both flexible two-way generation with and without pumping function

TRS	Condition	Flexible two-way (GWh)	Flexible two-way with pumping (GWh)
NWTL	Without WSL	195.04	222.68
	With WSL	194.11	221.77
	Difference	−0.48%	−0.41%
WSL	Without NWTL	219.46	254.06
	With NWTL	222.97	257.77
	Difference	1.6%	1.1%

**Fig. 7:**
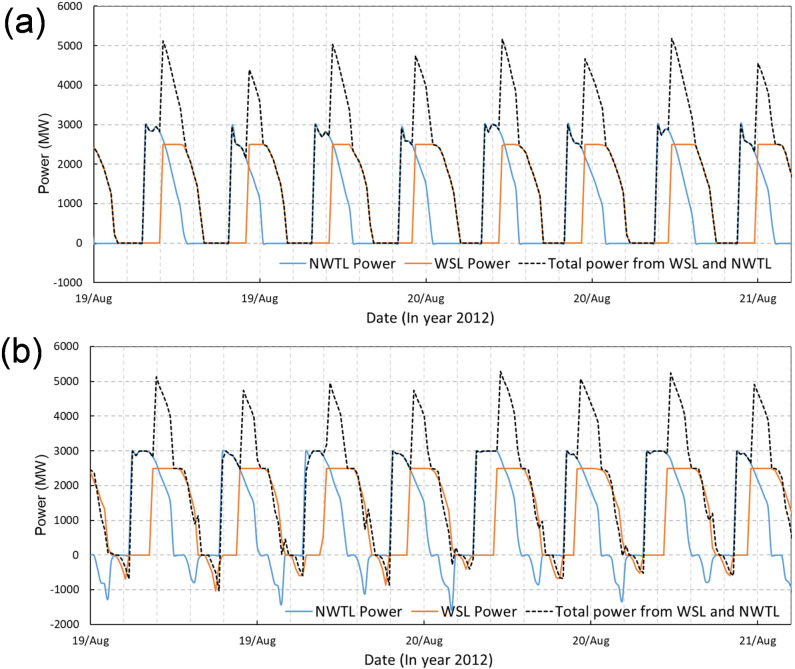
Power generation for the conjunctive operation of WSL and NWTL under **a** optimised two-way operation without pumping function; **b** optimised two-way operation with pumping function. The dash line indicates the combined power output

The tidal phase difference between the inner Bristol Channel and the Colwyn Bay is typically about 4 h, making WSL and NWTL a potentially effective multiple TRS system. Figure 7 gives the predicted power output from WSL and NWTL operating conjunctively. The duration with no power output was reduced from 2.5 to 3.75 h to approximately 1.5 h with two-way generation, and from 2.5 to 3 h to approximately 1.25 h with two-way generation including pumping.

### Hydrodynamic impact of individual operated SBL and WSL in the SEBC model

The comparison results for the individual and conjunctive operation of WSL and SBL were also not expected to be noticeably affected by the open boundary conditions. Thus, the conjunctive operation of SBL and WSL was simulated in the SEBC model, with the objective of focussing on the hydrodynamic changes within the Severn Estuary and Bristol Channel and with the simulations being undertaken with much higher computational efficiency.

**Fig. 8:**
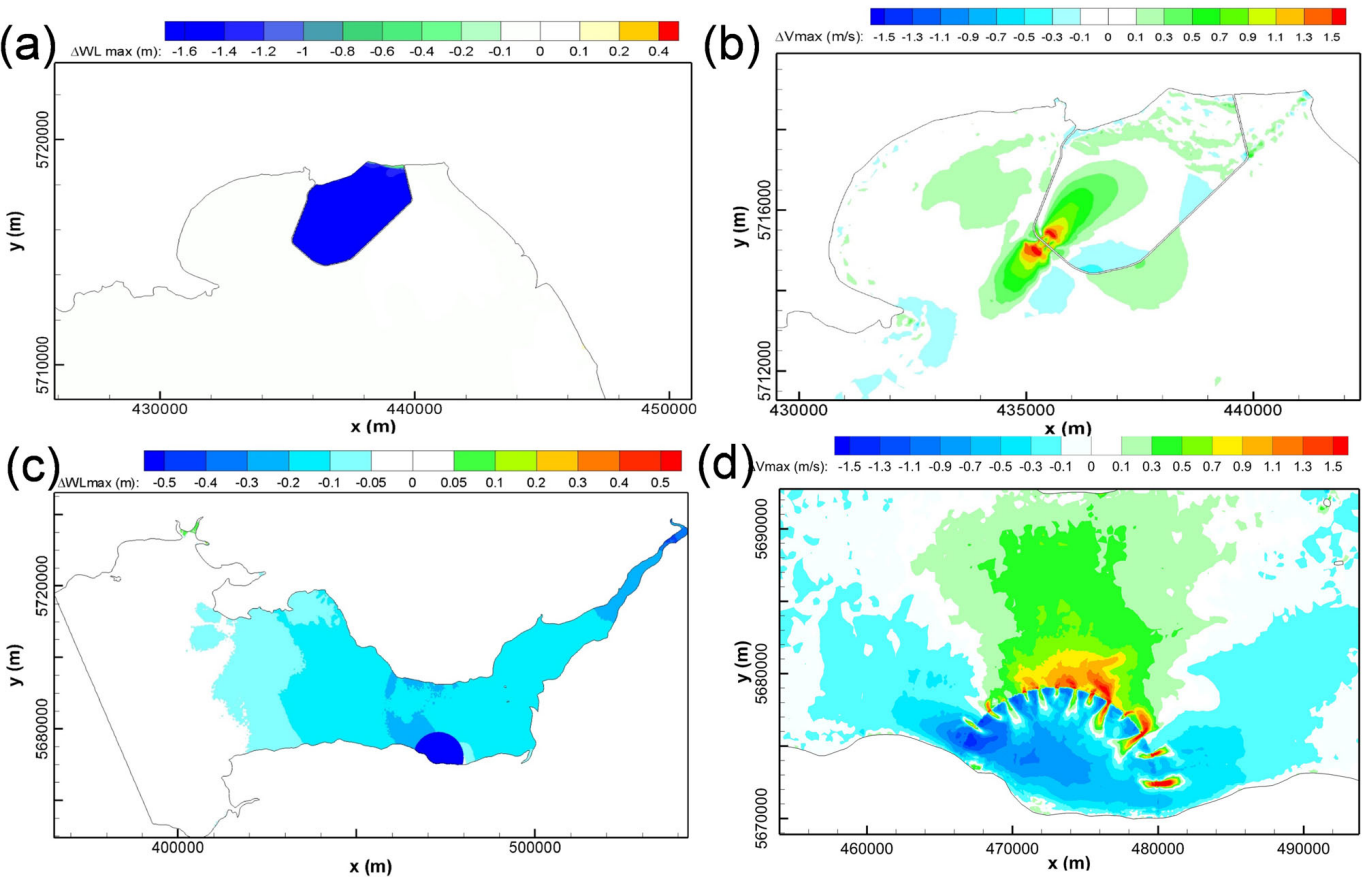
Cumulative impacts of individual operations of WSL and SBL on maximum water levels (left column) and maximum velocity (right column), as modelled by the SEBC model

WSL and SBL were operated individually in the SEBC model to predict their hydrodynamic impact on the maximum water levels and tidal currents, as shown in Fig. 8. The predictions show the influence of SBL on the maximum water level changes and these changes are generally confined within the Swansea Bay (Fig. 8(a)). Some very small water level oscillations were predicted outside of the SBL basin area due to the small impoundment area and low water storage volume, with a minor high water level decrease (less than 5 cm) on the western side of SBL. Similar to the maximum water level change, the influence of SBL on the current speeds was also generally limited within Swansea Bay. The wake downstream of the turbines and sluice gates resulted in a noticeable increase in the maximum velocity magnitude in the region of wake influence, with the peak velocity exceeding 1.5 m/s.

For the WSL modelling results obtained using the SEBC model, the operation generally increased the low water levels and decreased the high water levels in the Bristol Channel and Severn Estuary (Fig. 8(c)). The water level was predicted to drop by 0.05—0.2 m in the middle and inner Bristol Channel and by 0.2—0.3 m in the Severn Estuary. Except for the noticeable increase in the near-field maximum velocities in the turbine and sluice gate wakes, the maximum velocity in the inner Bristol Channel was predicted to increase by 0.25—0.75 m/s, whilst the corresponding maximum velocity decreased inside the lagoon and across most of the impounded water basin, particularly away from the turbine and sluice gate wakes.

### Effect of open boundary location on WSL modelling

In comparing the impact of WSL on the high water levels from the CS model (Fig. 4) and from the SEBC model (Fig. 8(a)), it was found that higher decreases in the peak high water level were predicted in the CS model. For example, a 20–30 cm high water level decrease was predicted in the inner Bristol Channel and Severn Estuary in the CS model, whilst only a small area in the SEBC model shows this variation. The high-water level variation extends to the outer Bristol Channel in the CS model, whilst it only reaches the central Bristol Channel in the SEBC model.

**Fig. 9:**
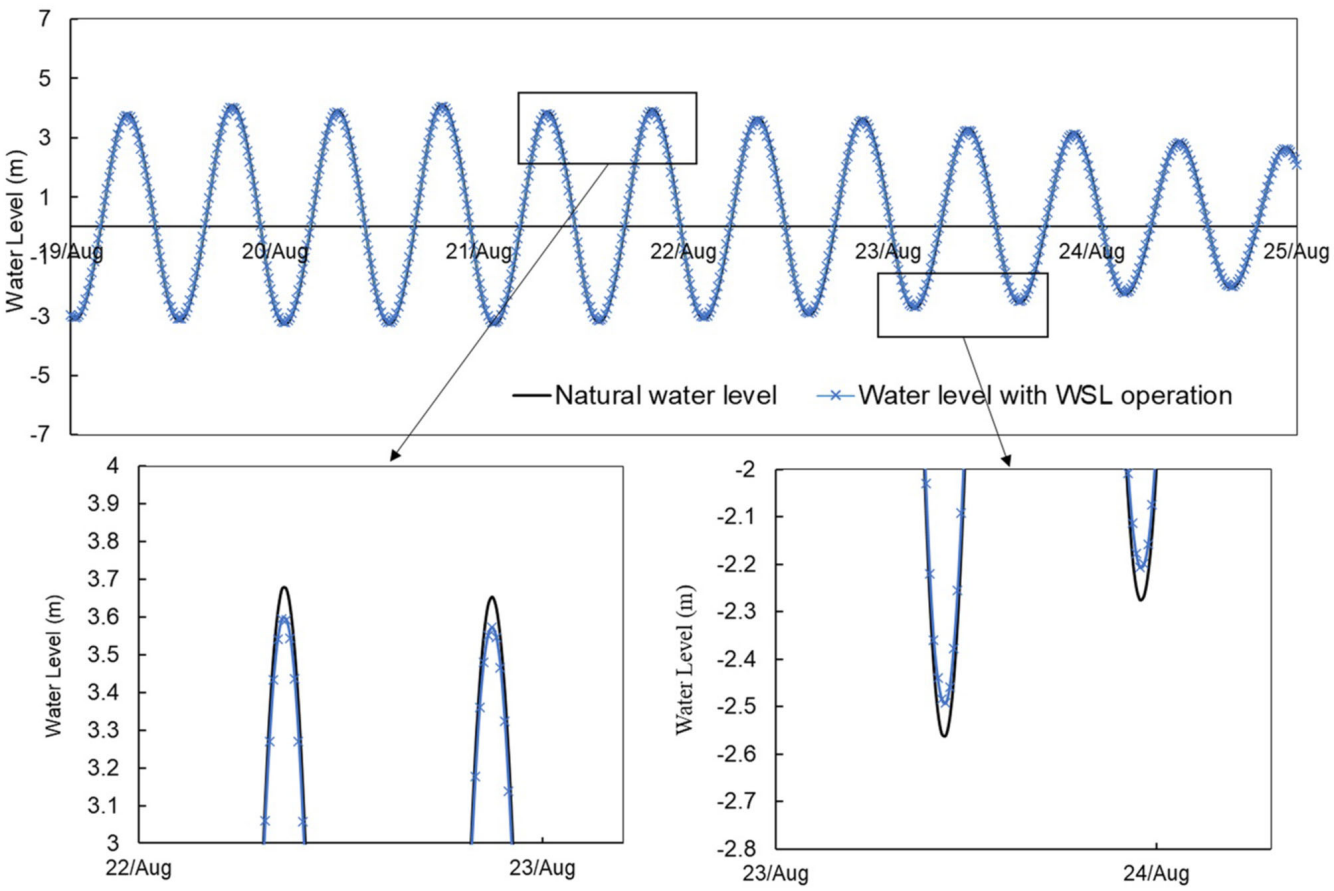
Changes in water level at the mouth of the Bristol Channel using the CS model before and after the operation of WSL

From these model results, it is clear that the operation of WSL has an influence on the open boundary conditions in the SEBC model. Figure 9 indicates that the WSL will increase the low-water level and decrease the high-water level by a few centimetres at the SEBC open boundary. These minor water level changes could make significant impact on electricity output with significant impact on feasibility of the schemes which highlight the importance of accurate representation of boundary condition in modelling TRSs. The annual electricity generation of WSL using the SEBC model is 5.73 TWh/yr and 6.62 TWh/yr for the optimised flexible generation without and with pumping, respectively, which were overpredicted by 6.0% and 5.6% compared with the predictions from the CS model.

### Hydrodynamic interactions between SBL and WSL

**Fig. 10:**
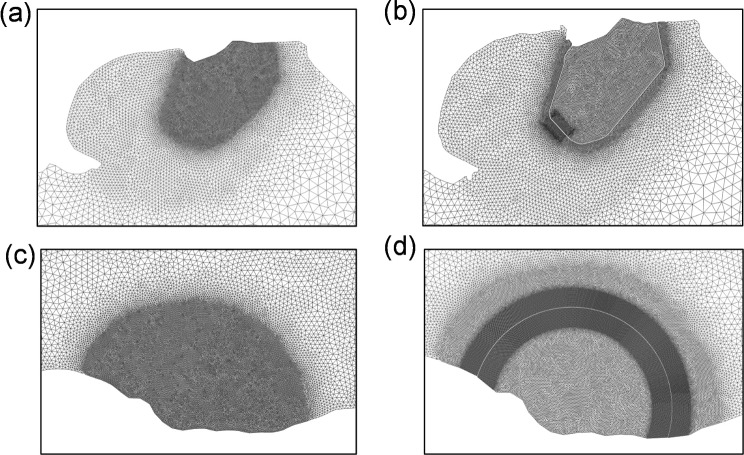
The regional grid resolutions for the model scenarios for: **a** Swansea Bay pre-lagoon; **b** Swansea Bay Lagoon; **c** West Somerset coast pre-lagoon; **d** West Somerset Lagoon

The conjunctive operation modelling of WSL and SBL follows the previous individual TRS simulation settings for each lagoon. To accurately assess the hydrodynamic impacts of the two lagoons on the hydrodynamics in the region, the mesh for the pre-lagoons and post-lagoon scenarios modelling was refined to include the same spatial distribution in the region away from the lagoons as presented in Fig. 10.

**Fig. 11:**
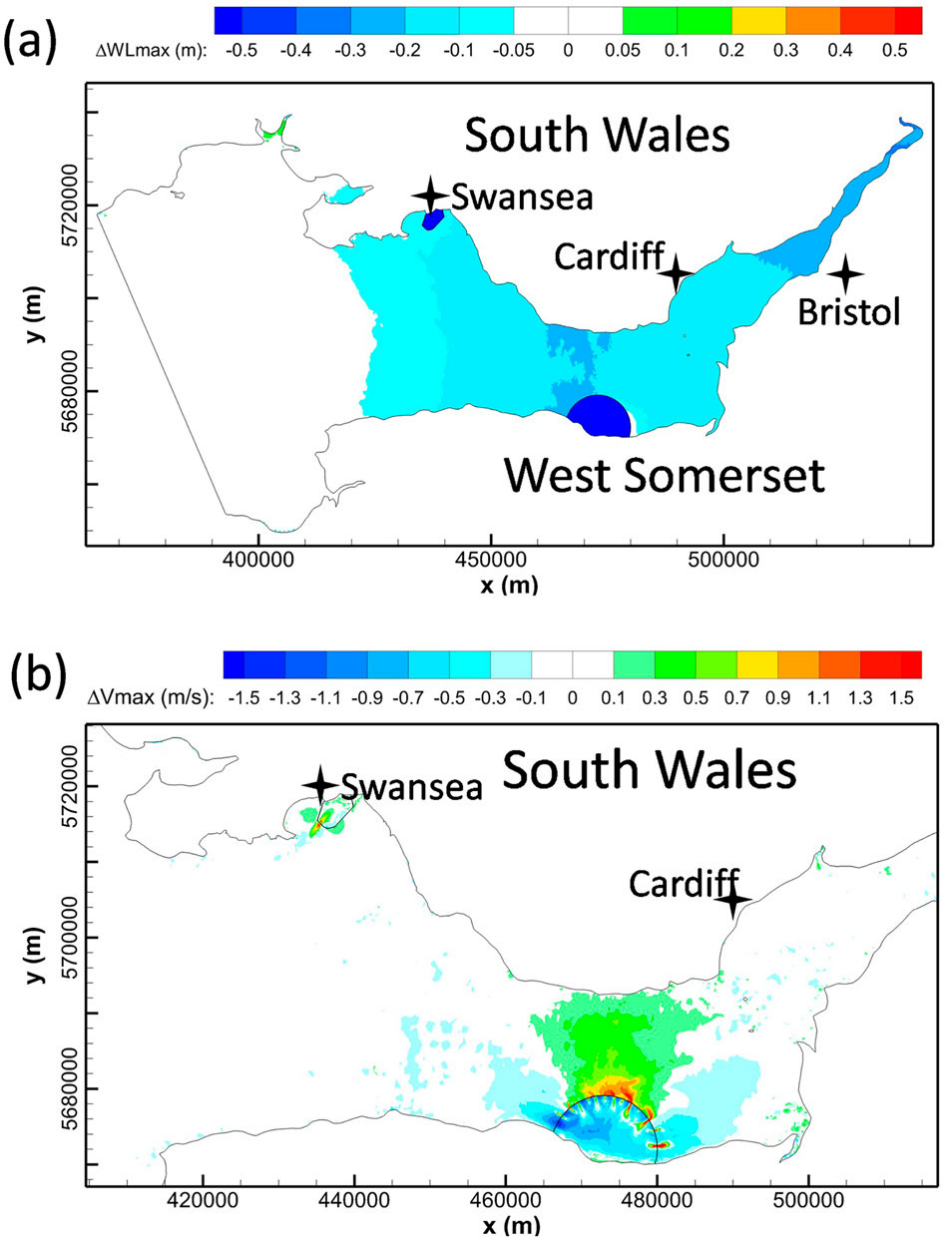
The cumulative impact of conjunctive operation of WSL and SBL on: **a** maximum water levels and **b** maximum velocity

**Fig. 12:**
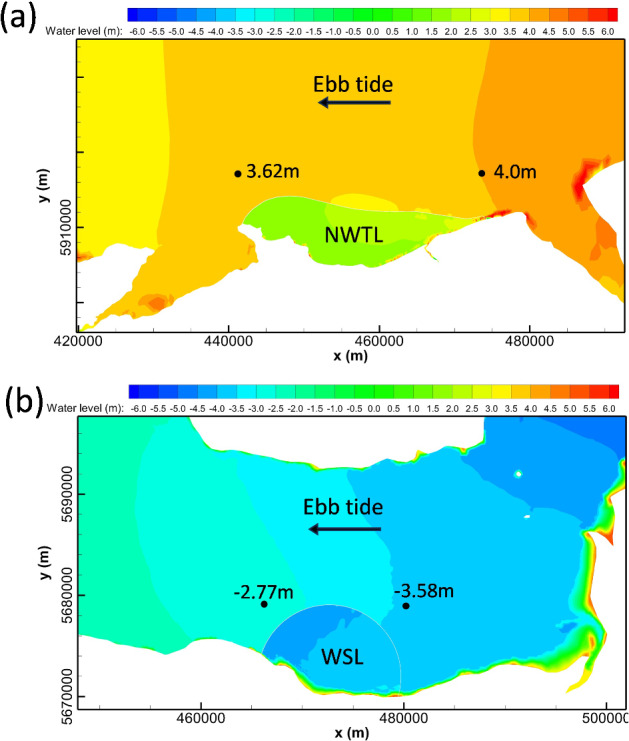
The significant water level difference along the embankment of: **a** North Wales Tidal Lagoon; **b** West Somerset Lagoon, with marked water levels on both sides of the lagoon structures

**Fig. 13:**
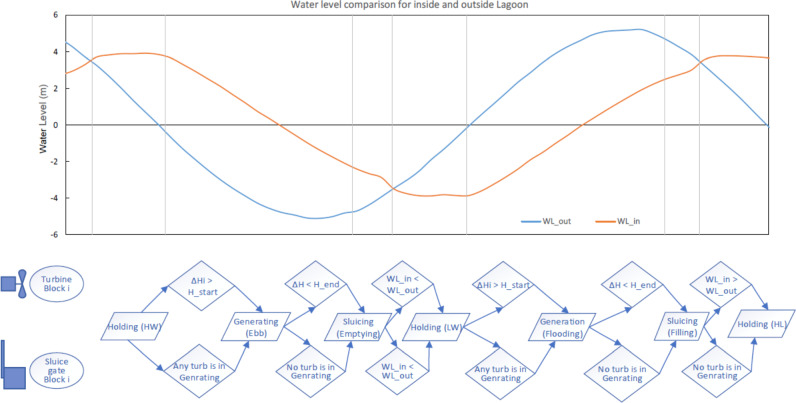
Flowchart illustrating the two-way operational mode, where each turbine and sluice gate block is managed by an independent decision-making system. The upper panel shows the water level comparison between the inside and outside of the lagoon impoundment, corresponding to the various stages of lagoon operation. (HW/LW = High/Low water level, ∆Hi = water level difference across the hydraulic structure, H_start = desired head difference to initiate turbine operation (m), H_end = minimum head difference for end of turbine operation (m))

**Fig. 14:**
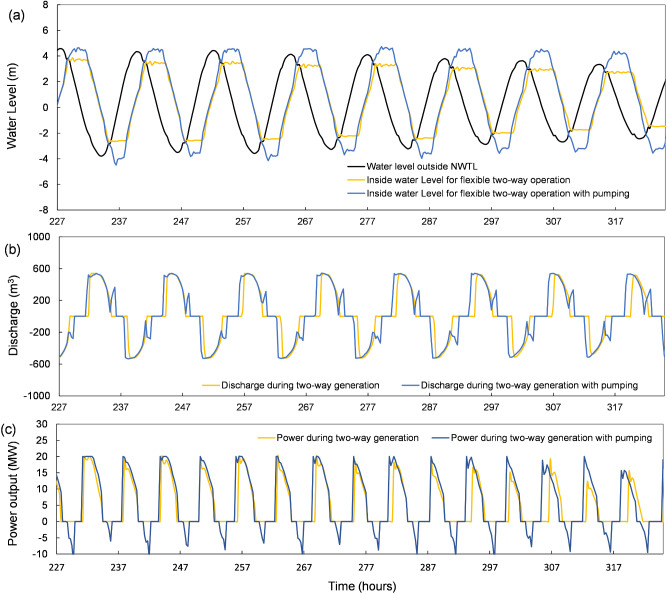
Predicted outputs for the North Wales Tidal Lagoon including: **a** water level variations downstream and upstream of the impoundments, **b** discharge variations through a single turbine, and **c** power output for a single turbine. The yellow line indicates the flexible two-way generation scheme, and the blue line indicates the flexible two-way operation with pumping

**Fig. 15:**
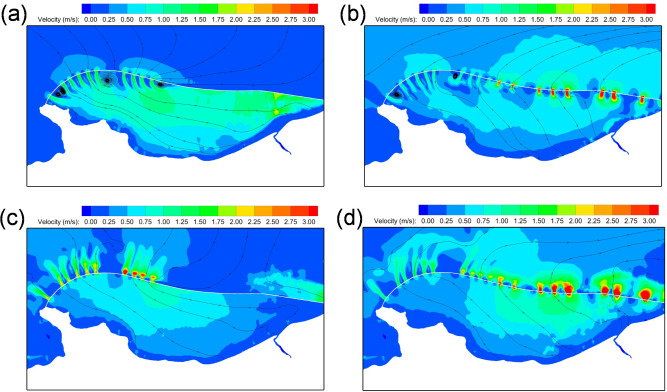
Typical instantaneous flow patterns and velocity magnitude during two-way operation of NWTL, and the flow direction is indicated with black streamlines, for different operation stages: **a** flood generation at the high sea water level, **b** filling phase at the end of flood generation whilst the sea water level outside lagoon is still higher than inside, **c** ebb generation at the low sea water level, **d** emptying phase at the end of ebb generation whilst the sea water level inside lagoon is still higher than outside

**Fig. 16:**
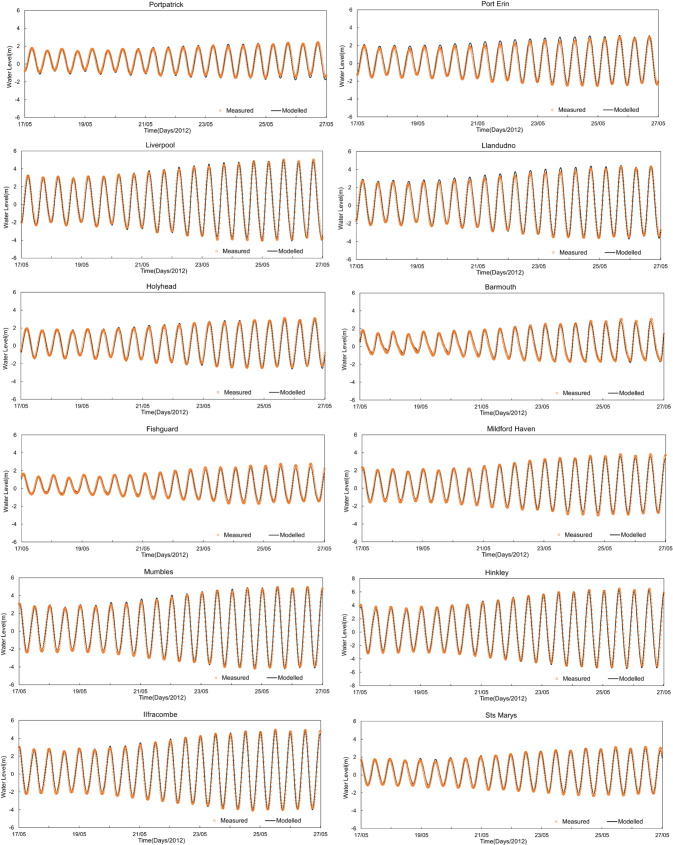
Water level data comparison between measured and predicted values at BODC tidal gauge sites

**Fig. 17:**
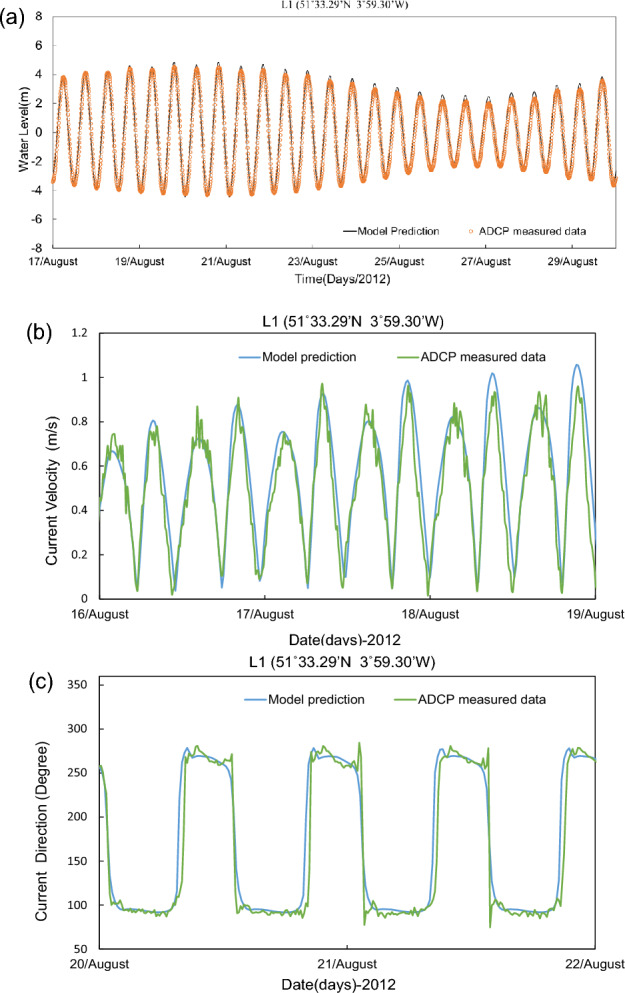
Comparison of CS Model predicted and ADCP measured data in Swansea Bay at the ADCP L1 measuring site: **a** water levels; **b** tidal current speeds; **c** tidal current directions

Figure 11 shows the high water levels and the maximum velocity changes in the Bristol Channel with the joint operation of WSL and SBL. By comparing the predicted individual operation for WSL (Fig. 8(c)) and SBL (Fig. 8(a)) with the conjunctive operation results (Fig. 11), it can be concluded that the maximum water level changes are similar between the scenarios for conjunctive operation and the WSL individual operation. However, there are slight differences in the high-water level decrease region, e.g. the 0.2—0.3 m high-water level decrease zone in the Severn Estuary, which extends slightly more seawards for the conjunctive operation predictions. The impact of individual operation and conjunctive operation of SBL and WSL on the maximum velocities is illustrated in Fig. 8(b), Fig. 8(d), and Fig. 11(b). With regard to the maximum velocity magnitude changes, negligible interactions are observed between WSL and SBL. These results suggest that despite these lagoons being relatively near to one another, each lagoon independently dominates the velocity structure around the respective lagoon structure.

It is noted that the operation of SBL has negligible influence on WSL power generation (Table 3), whilst the existence of WSL reduces the electricity output for SBL by about 3.7%. The variations in power output concur with the hydrodynamic variations that WSL reduces the tidal range slightly in Swansea Bay by a few centimetres (Fig. 8(c)), but SBL hardly influences the water level changes in far-field such as the West Somerset coast.

**Table 3:** Electricity outputs of WSL and SBL for individual and conjunctive operation in one entire tidal cycle, for flexible two-way generation only

TRS	Condition	Flexible two-way (GWh)
WSL	Without SBL	233.48
	With SBL	231.84
	Difference	−0.76%
SBL	Without WSL	23.74
	With WSL	22.87
	Difference	−3.7%

## Discussion

Whilst the impoundment area of NWTL is 47% larger than WSL, the hydrodynamic impact from WSL travels further due to the funnelling effect of the wedge-shaped the Bristol Channel and the impacts of tidal resonance (Ma and Adcock 2020). The combined power output from conjunctive operation of WSL and NWTL shows great potential in proving near-continuous tidal energy. However, it is important to address the issue of the overlapping operation period of WSL and NWTL as it leads to a sudden surge in power outputs. This aspect requires careful consideration, particularly in terms of grid management and ensuring a stable and reliable power supply.

Although the hydrodynamic impacts of WSL were generally consistent between the CS and SEBC models, the predicted results showed that the impact of WSL on the water levels extended to the outer Bristol Chanel in the CS model, with over a 10 cm decrease in the tidal range along the location of the open seaward boundary of the SEBC model. This observation is consistent with previous research findings where the effects of the open boundary location on model predictions for a Severn Barrage showed that the area where water levels were affected varied when an inappropriate boundary location was selected (Zhou et al. 2014). The comparison between Fig. 4 and Fig. 8 shows that although there is a detectable difference in the maximum water levels in the outer Bristol Channel between the two models, the range and extent of the maximum water level changes are similar in the Severn Estuary and the inner Bristol Channel. Furthermore, this study focussed on exploring the potential interaction between tidal lagoons, which is less influenced by the open boundary location. Thus, in the opinion and experience of the authors, the SEBC model is sufficient for the purposes of this study to model and assess the impact of WSL and to predict the impact when considered in conjunctive operation with SBL. However, for other TRS studies, such as power prediction, then a more precise open boundary condition may be needed, either by preferably extending the model domain to the Continental Shelf or alternatively theoretically modifying the open boundary characteristics (Adcock et al. 2015).

There exist certain uncertainties with respect to the far-field hydrodynamic impact of lagoon operations, which fall within the bounds of model validation error. Examples include the mitigated water level changes in Cardigan Bay as the results of conjunctive operation of WSL and NWTL (Fig. 3 and Fig. 5), the slight velocity changes observed in Liverpool Bay between pre- and post-WSL operations (as shown in Fig. 4), as well as the alterations in tidal range noted in a different lagoon-proposed region (as presented in Table 1 and Fig. 6). Although the same numerical settings were applied throughout different modelling scenarios, and the potential prediction deviation that could originate from the mesh resolutions has been excluded by maintaining the same mesh resolutions, the associated hydrodynamic variations are relatively minor and fall within the limits of model validation. For instance, this research shows that the sea water level influence of WSL and NWTL in Cardigan Bay is only a few centimetres, but the validation results indicate that the maximum water level deviation at the nearest BODC gauge (Barmouth) can be of the order of decimetres. Given the minor far-field hydrodynamic changes observed, it is important to approach these results with extra care and therefore further investigation is required.

The scope of this research reveals the minor interaction between conjunctive operation of WSL and NWTL, as well as WSL and SBL. The interactions between lagoons are dependent on factors, such as lagoon scale, location, tidal phase and others, therefore a general conclusion could not be obtained. However, it is worth considering that the outcomes of this research may be constrained by the specific modelling scenarios employed. It is recommended to conduct further studies on the conjunctive operation of tidal range schemes, encompassing a diverse range of specific and generalised scenarios.

## Conclusions

This paper investigates the conjunctive and individual operations and potential interactions of three tidal lagoons. Two hydrodynamic models, the Seven Estuary and Bristol Channel (SEBC) model and the Continental Shelf (CS) model, were developed using TELEMAC-2D to simulate the individual and conjunctive operation of North Wales Tidal Lagoon (NWTL), West Somerset Lagoon (WSL) and Swansea Bay Lagoon (SBL).

The interaction study between lagoon conjunctive operations showed that the hydrodynamic impacts in the vicinity of the schemes were dominated by the respective lagoons. In the conjunctive operation of WSL and NWTL, slight superimposed effects were found in the far-field, i.e. Cardigan Bay, where the maximum water level oscillations from individual operations of each lagoon were mitigated. For the conjunctive operation of WSL and SBL, it was predicted that the 0.2—0.3 m high water level decrease zone in the Severn Estuary extended slightly more towards the seaward boundary. However, the influence of the lagoon interactions was generally found to be small on the power output predictions for each lagoon individually.

It was predicted that the period of no power generation for the individual operation of the WSL and NWTL was reduced from 2.5 to 3.75 h to less than 1.5 h when the lagoons were considered to operate conjunctively. This meant that when operated conjunctively, the combined lagoons were able to provide power for close to the full tidal cycle. This opportunity for generating almost continuous tidal power offsets any disadvantage in terms of a reduction in power generation from the operation of a single TRS influenced by the presence of another one nearby.

The effect of open boundary location on lagoon modelling has been investigated by comparing the performance of WSL in the CS and SEBC model. The hydrodynamic impact of WSL is generally consistent between CS and SEBC models. However, the oscillation of maximum water level results from the operation of WSL extended to the outer Bristol Channel in the CS model, suggesting the existence of the influence of WSL operation on SEBC model open boundary.

## Electronic supplementary material

Below is the link to the electronic supplementary material.

Supplementary file1 (DOCX 11812 KB)

## Data Availability

Data used to produce the results will be available on request.

## Appendix

See Figs. 12, 13, 14, 15, 16, 17. See Table 4.

**Table 4:** Validation statistics of water level data obtained from BODC gauges

Site	R^2^	RMSE (m)
Portpatrick	0.971	0.174
Port Erin	0.968	0.249
Liverpool	0.985	0.281
Llandudno	0.987	0.233
Holyhead	0.987	0.158
Barmouth	0.964	0.216
Fishguard	0.970	0.177
Milford Haven	0.978	0.250
Mumbles	0.975	0.374
Hinkley	0.967	0.553
Ilfracombe	0.979	0.336
St Mary’s	0.984	0.171

## References

[CR1] Adcock TAA, Draper S, Nishino T (2015) Tidal power generation – a review of hydrodynamic modelling. Proc Inst Mech Eng A J Power Energy 229:755–771. 10.1177/0957650915570349

[CR2] Ahmadian R, Falconer RA, Lin B (2010) Hydro-environmental modelling of proposed Severn barrage, UK. Proc Inst Civ Eng Energy 163:107–117. 10.1680/ener.2010.163.3.107

[CR3] Ahmadian R, Olbert AI, Hartnett M, Falconer RA (2014) Sea level rise in the Severn Estuary and Bristol Channel and impacts of a Severn Barrage. Comput Geosci 66:94–105. 10.1016/j.cageo.2013.12.011

[CR4] Angeloudis A, Ahmadian R, Falconer R, Bockelmann-Evans B (2015) Combined potential and impacts of tidal lagoons along the North Wales coast. In: 2015 Proceedings of the 36th IAHR World Congress, Hague, Netherlands, pp 7277–7284. https://www.iahr.org/library/infor?pid=7981

[CR5] Angeloudis A, Falconer RA (2017) Sensitivity of tidal lagoon and barrage hydrodynamic impacts and energy outputs to operational characteristics. Renew Energy 114:337–351. 10.1016/j.renene.2016.08.033

[CR6] Angeloudis A, Falconer RA, Bray S, Ahmadian R (2016) Representation and operation of tidal energy impoundments in a coastal hydrodynamic model. Renew Energy 99:1103–1115. 10.1016/j.renene.2016.08.004

[CR7] Angeloudis A, Kramer SC, Avdis A, Piggott MD (2018) Optimising tidal range power plant operation. Appl Energy 212:680–690. 10.1016/j.apenergy.2017.12.052

[CR8] Bourban S, Durand N, Turnbull M, Wilson S, Cheeseman S (2012) Coastal shelf model of northern European waters to inform tidal power industry decisions. In: 2012 Proceedings of the XIXth TELEMAC-MASCARET User Conference, Oxford, UK, pp 143–150. TELEMAC-MASCARET_Proceedings-2012-R1.pdf

[CR9] Cornett A, Cousineau J (2011) Hydrodynamic impacts due to tidal power lagoons in the Upper Bay of Fundy, Canada. In: 2011 Proceeding of European Wave and Tidal Energy Conf (EWTEC), Southampton, UK.

[CR11] Cornett A, Cousineau J, Nistor I (2013) Assessment of hydrodynamic impacts from tidal power lagoons in the Bay of Fundy. Int J Mar Energy 1:33–54. 10.1016/j.ijome.2013.05.006

[CR10] Cho YS, Lee JW, Jeong W (2012) The construction of a tidal power plant at Sihwa Lake, Korea. Energy Sources Part A Recover Util Environ Eff 34:1280–1287. 10.1080/15567030903586055

[CR12] Čož N, Ahmadian R, Falconer RA (2019) Implementation of a full momentum conservative approach in modelling flow through tidal structures. Water 11:1917. 10.3390/w11091917

[CR13] Dushaw BD, Egbert GD, Worcester PF, Cornuelle BD, Howe BM, Metzger K (1997) A TOPEX/POSEIDON global tidal model (TPXO.2) and barotropic tidal currents determined from long-range acoustic transmissions. Prog Oceanogr 40:337–367. 10.1016/S0079-6611(98)00008-1

[CR14] Falconer RA, Guo B, Ahmadian R (2020) Coastal reservoirs and their potential for urban regeneration and renewable energy supply. In: Sitharam TG, Yang SQ, Falconer RA, Sivakumar M, Jones B, Kolathayar S, Sinpoh L (eds) Sustainable water resource development using coastal reservoirs. pp 143–172.

[CR15] Guo B, Ahmadian R, Evans P, Falconer RA (2020) Studying the wake of an island in a macro-tidal estuary. Water 12:1225. 10.3390/w12051225

[CR16] Guo B, Ahmadian R, Falconer RA (2021) Refined hydro-environmental modelling for tidal energy generation: West Somerset Lagoon case study. Renew Energy 179:2104–2123. 10.1016/j.renene.2021.08.034

[CR17] Hanousek N, Ahmadian R, Lesurf E (2023) Providing distributed electrical generation through retrofitting disused docks as tidal range energy schemes. Renew Energy 2023:119149. 10.1016/j.renene.2023.119149

[CR18] Haverson D, Bacon J, Smith HCM, Venugopal V, Xiao Q (2017) Cumulative impact assessment of tidal stream energy extraction in the Irish Sea. Ocean Eng 137:417–428. 10.1016/j.oceaneng.2017.04.003

[CR19] Hervouet JM (2007) Hydrodynamics of free surface flows: modelling with the finite element method. John Wiley & Sons Ltd, West Sussex, England.

[CR20] Hooper T, Austen M (2013) Tidal barrages in the UK: ecological and social impacts, potential mitigation, and tools to support barrage planning. Renew Sustain Energy Rev 23:289–298. 10.1016/j.rser.2013.03.001

[CR21] Ji H, Pan S, Chen S (2020) Impact of river discharge on hydrodynamics and sedimentary processes at Yellow River Delta. Mar Geol 425:106210. 10.1016/j.margeo.2020.106210

[CR22] Kadiri M, Ahmadian R, Bockelmann-Evans B, Falconer RA, Kay D (2014) An assessment of the impacts of a tidal renewable energy scheme on the eutrophication potential of the Severn Estuary, UK. Comput Geosci 71:3–10. 10.1016/j.cageo.2014.07.018

[CR23] Lewis M, Neill SP, Robins PE, Hashemi MR (2015) Resource assessment for future generations of tidal-stream energy arrays, and comparison of TPXO and FES2012. Energy 83:403–415. 10.1016/j.energy.2015.02.038

[CR24] Ma Q, Adcock TAA (2020) Modification of tidal resonance in the Severn Estuary by a barrage and lagoon. J Ocean Eng Mar Energy 6:171–181. 10.1007/s40722-020-00166-8

[CR25] MacKay DJC (2007) Enhancing electrical supply by pumped storage in tidal lagoons. Cavendish Laboratory, University of Cambridge. https://democracy.kent.gov.uk/documents/s11499/Lagoons.pdf.

[CR26] Mackie L, Coles D, Piggott M, Angeloudis A (2020) The potential for tidal range energy systems to provide continuous power: a UK case study. J Mar Sci Eng 8:780. 10.3390/jmse8100780

[CR27] Neill SP, Angeloudis A, Robins PE, Walkington I, Ward SL, Masters I et al (2018) Tidal range energy resource and optimization – past perspectives and future challenges. Renew Energy 127:763–778. 10.1016/j.renene.2018.05.007

[CR28] North Wales Tidal Energy Ltd (2020) North Wales presents a world-class site for a tidal lagoon. https://www.northwalestidalenergy.com/concept.

[CR29] Rainey RCT (2009) The optimum position for a tidal power barrage in the Severn estuary. J Fluid Mech 636:497–507. 10.1017/S0022112009991443

[CR30] Rtimi R, Sottolichio A, Tassi P (2021) Hydrodynamics of a hyper-tidal estuary influenced by the world’s second largest tidal power station (Rance estuary, France). Estuar Coast Shelf Sci 250:107143. 10.1016/j.ecss.2020.107143

[CR31] Serhadlıoğlu S, Adcock TAA, Houlsby GT, Draper S, Borthwick AG (2013) Tidal stream energy resource assessment of the Anglesey Skerries. Int J Mar Energy 3:98–111. 10.1016/j.ijome.2013.11.014

[CR32] Todeschini G (2017) Review of tidal lagoon technology and opportunities for integration within the UK energy system. Inventions 2:14. 10.3390/inventions2030014

[CR33] Vazquez A, Iglesias G (2015) LCOE (levelised cost of energy) mapping: a new geospatial tool for tidal stream energy. Energy 91:192–201. 10.1016/j.energy.2015.08.012

[CR34] Waters S, Aggidis G (2016) Tidal range technologies and state of the art in review. Renew Sustain Energy Rev 59:514–529. 10.1016/j.rser.2015.12.347

[CR35] Wilson, S., Bourban, S., & Couch, S. (2012) Understanding the interactions of tidal power projects across the UK Continental Shelf *Proceedings of the 4th International Conference on Ocean Energy, Dublin, Ireland*.

[CR36] Wilson S, Bourban S, Couch S (2012) Understanding the interactions of tidal power projects across the UK Continental Shelf. In: 2012 Proceeding of 4th Int Conf Ocean Energy, Dublin, Ireland.

[CR37] Wolf J, Walkington IA, Holt J, Burrows R (2009) Environmental impacts of tidal power schemes. Marit Eng 162:165–177. 10.1680/maen.2009.162.4.165

[CR38] Xia J, Falconer RA, Lin B (2010a) Hydrodynamic impact of a tidal barrage in the Severn Estuary, UK. Renew Energy 35:1455–1468. 10.1016/j.renene.2009.12.009

[CR39] Xia J, Falconer RA, Lin B (2010b) Impact of different tidal renewable energy projects on the hydrodynamic processes in the Severn Estuary, UK. Ocean Model 32:86–104. 10.1016/j.ocemod.2009.11.002

[CR40] Xue J, Ahmadian R, Jones O (2020) Genetic algorithm in tidal range schemes’ optimisation. Energy 200:117496. 10.1016/j.energy.2020.117496

[CR41] Zhou J, Pan S, Falconer RA (2014) Effects of open boundary location on the far-field hydrodynamics of a Severn barrage. Ocean Model 73:19–29. 10.1016/j.ocemod.2013.10.006

