# Supplementary material for: Individual and conjunctive operation of tidal lagoons along the west coast of the UK
Source: J Ocean Eng Mar Energy. 2025 Sep 1;12(1):1–21. doi: 10.1007/s40722-025-00420-x (PMC12835122; doi:10.1007/s40722-025-00420-x)
Supplement: Supplementary file 1 — Supplementary file1 (DOCX 11812 KB) [file 40722_2025_420_MOESM1_ESM.docx]

# 9. Appendix


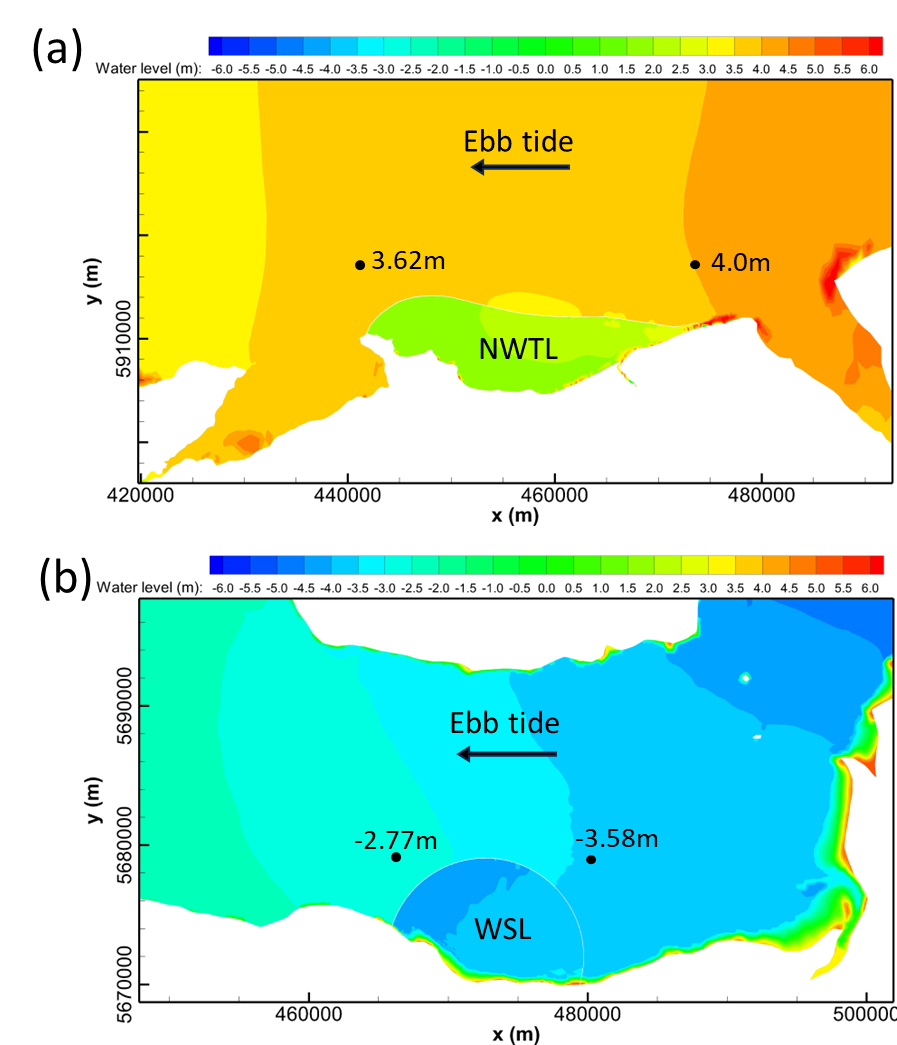


Fig. A1: The significant water level difference along the embankment of: (a) North Wales Tidal Lagoon; (b) West Somerset Lagoon, with marked water levels on both sides of the lagoon structures.


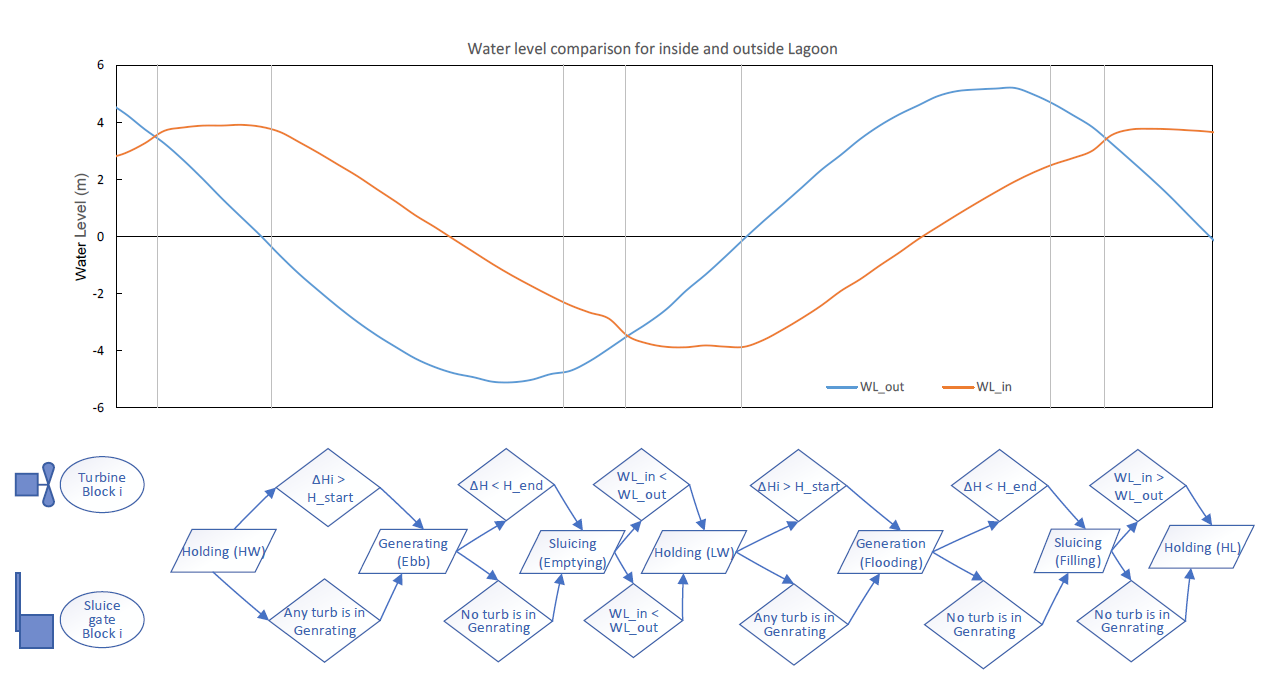


Fig. A2: Flowchart illustrating the two-way operational mode, where each turbine and sluice gate block is managed by an independent decision-making system. The upper panel shows the water level comparison between the inside and outside of the lagoon impoundment, corresponding to the various stages of lagoon operation. (HW/LW = High/Low water level, ∆Hi = water level difference across the hydraulic structure, H_start = desired head difference to initiate turbine operation (m), H_end = minimum head difference for end of turbine operation (m) )


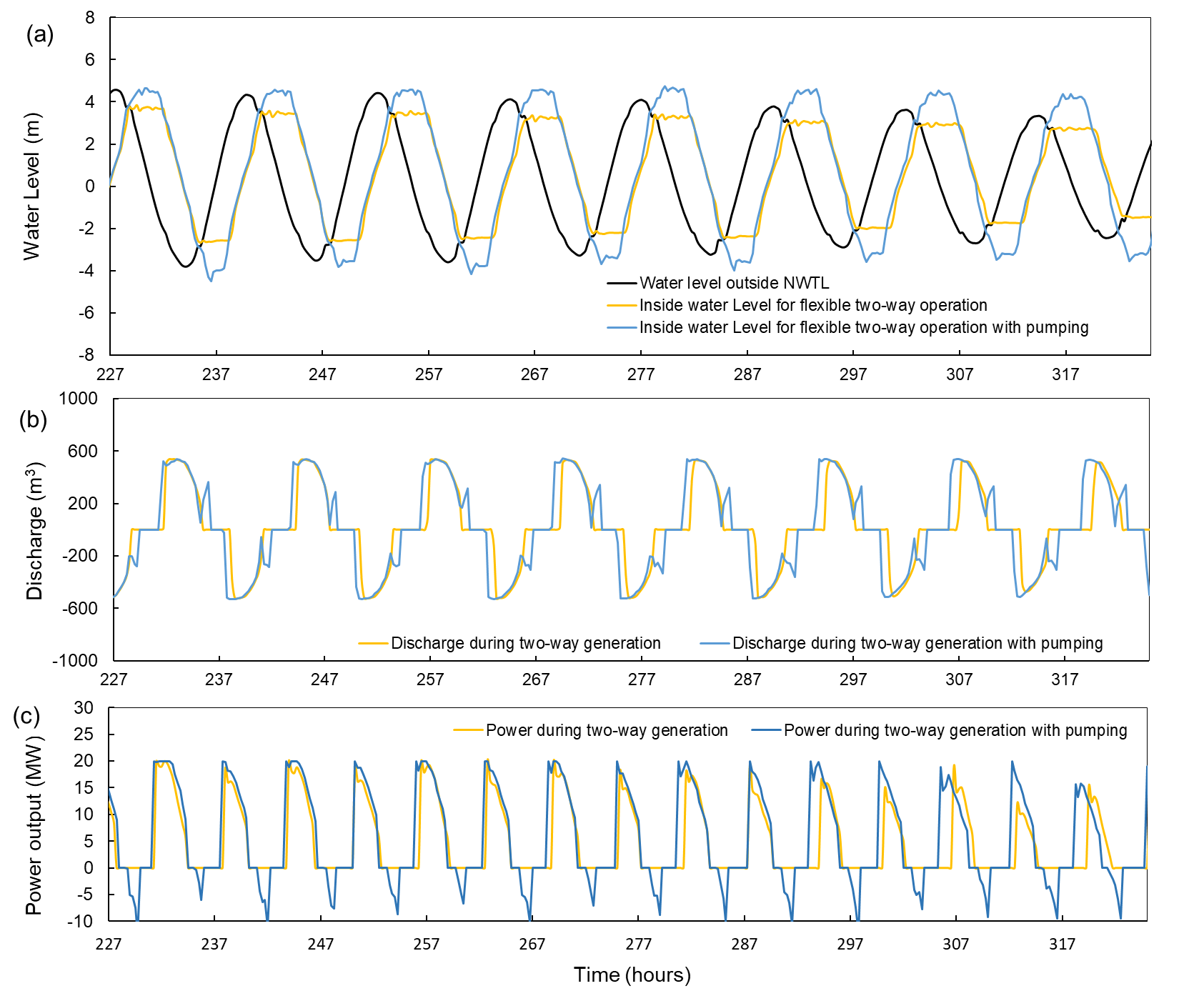


Fig. A3: Predicted outputs for the North Wales Tidal Lagoon including: (a) water level variations downstream and upstream of the impoundments, (b) discharge variations through a single turbine, and (c) power output for a single turbine. The yellow line indicates the flexible two-way generation scheme, and the blue line indicates the flexible two-way operation with pumping.


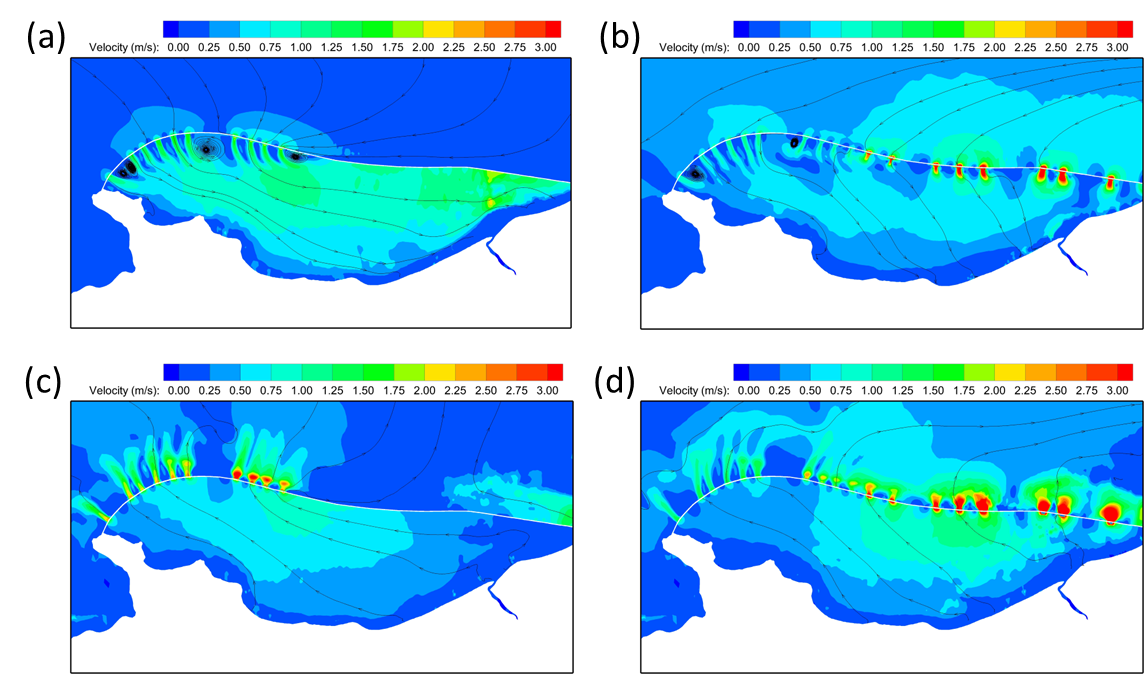


Fig. A4: Typical instantaneous flow patterns and velocity magnitide during two-way operation of NWTL, and the flow direction is indicated with black streamlines, for different operation stages: (a) flood generation at the high sea water level, (b) filling phase at the end of flood generation while the sea water level outside lagoon is still higher than inside, (c) ebb generation at the low sea water level, (d) emptying phase at the end of ebb generation while the sea water level inside lagoon is still higher than outside.

Table A1: Validation statistics of water level data obtained from BODC gauges.

| Site | R^2^ | RMSE (m) |
| --- | --- | --- |
| Portpatrick | 0.971 | 0.174 |
| Port Erin | 0.968 | 0.249 |
| Liverpool | 0.985 | 0.281 |
| Llandudno | 0.987 | 0.233 |
| Holyhead | 0.987 | 0.158 |
| Barmouth | 0.964 | 0.216 |
| Fishguard | 0.970 | 0.177 |
| Milford Haven | 0.978 | 0.250 |
| Mumbles | 0.975 | 0.374 |
| Hinkley | 0.967 | 0.553 |
| Ilfracombe | 0.979 | 0.336 |
| St Mary’s | 0.984 | 0.171 |

Fig. A5: Water level data comparison between measured and predicted values at BODC tidal gauge sites.

Fig.A6: Comparison of CS Model predicted and ADCP measured data in Swansea Bay at the ADCP L1 measuring site: (a) water levels; (b) tidal current speeds; (c) tidal current directions.
